# Assessment of attenuation correction for myocardial PET imaging using combined PET/MRI

**DOI:** 10.1007/s12350-017-1118-2

**Published:** 2017-11-22

**Authors:** Martin Lyngby Lassen, Sazan Rasul, Dietrich Beitzke, Marie-Elisabeth Stelzmüller, Jacobo Cal-Gonzalez, Marcus Hacker, Thomas Beyer

**Affiliations:** 1 QIMP Group, Center for Medical Physics and Biomedical Engineering, General Hospital Vienna, Medical University of Vienna, 1090 Vienna, Austria; 2 Division of Nuclear Medicine, Department of Biomedical Engineering and Image-guided Therapy, Medical University of Vienna, Vienna, Austria; 3 Division of Cardiovascular and Interventional Radiology, Department of Biomedical Engineering and Image-guided Therapy, Medical University of Vienna, Vienna, Austria; 4 Department of Cardiac Surgery, Medical University of Vienna, Vienna, Austria

**Keywords:** Attenuation correction, cardiac PET, artifacts, PET/MR

## Abstract

**Objective:**

To evaluate the frequency of artifacts in MR-based attenuation correction (AC) maps and their impact on the quantitative accuracy of PET-based flow and metabolism measurements in a cohort of consecutive heart failure patients undergoing combined PET/MR imaging.

**Methods:**

Myocardial viability studies were performed in 20 patients following a dual-tracer protocol involving the assessment of myocardial perfusion (^13^N-NH_3_: 813 ± 86 MBq) and metabolism (^18^F-FDG: 335 ± 38 MBq). All acquisitions were performed using a fully-integrated PET/MR system, with standard DIXON-attenuation correction (DIXON-AC) mapping for each PET scan. All AC maps were examined for spatial misalignment with the emission data, total lung volume, susceptibility artifacts, and tissue inversion (TI). Misalignment and susceptibility artifacts were corrected using rigid co-registration and retrospective filling of the susceptibility-induced gaps, respectively. The effects of the AC artifacts were evaluated by relative difference measures and perceived changes in clinical interpretations.

**Results:**

Average respiratory misalignment of (7 ± 4) mm of the PET-emission data and the AC maps was observed in 18 (90%) patients. Substantial changes in the lung volumes of the AC maps were observed in the test–retest analysis (ratio: 1.0 ± 0.2, range: 0.8-1.4). Susceptibility artifacts were observed in 10 (50%) patients, while six (30%) patients had TI artifacts. Average differences of 14 ± 10% were observed for PET images reconstructed with the artifactual AC maps. The combined artifact effects caused false-positive findings in three (15%) patients.

**Conclusion:**

Standard DIXON-AC maps must be examined carefully for artifacts and misalignment effects prior to AC correction of cardiac PET/MRI studies in order to avoid misinterpretation of biased perfusion and metabolism readings from the PET data.

**Electronic supplementary material:**

The online version of this article (10.1007/s12350-017-1118-2) contains supplementary material, which is available to authorized users.

## Introduction

Myocardial perfusion and metabolism imaging has become routine in nuclear medicine using positron emission tomography/computed tomography (PET/CT) systems.^1^,^2^ Recently, the introduction of combined PET/ magnetic resonance (PET/MR) Imaging systems has opened new possibilities for performing multi-parametric assessments of myocardial perfusion and viability.^3^

Myocardial perfusion imaging (MPI) can be used for the diagnosis of coronary artery disease (CAD).^4^,^5^ However, acquisitions of both MPI and myocardial metabolism imaging mandates accurate attenuation correction (AC) of the PET-emission data.^6^–^8^ Traditionally, cardiovascular imaging has been performed in PET-only and PET/CT systems by relying on AC maps obtained from a rotating transmission source or CT measurement, respectively.^9^–^11^ In combined PET/MR systems, these options are not available and alternative solutions have to be found. Thus, segmented MR-based AC (MR-AC) maps have been chosen as the current AC method-of-choice for integrated PET/MR imaging. Transformation from MR-images into AC maps are based on segmentation algorithms, which segment the images into four tissue types, with each tissue classification assigned to a vendor-specific attenuation (ATN) value.^12^ In the Siemens Biograph mMR system, the segmented AC map is based on a DIXON-VIBE sequence, composed of in- and opposed-phase images that are recomposed into fat and water images, and segmented into four tissue types (fat, soft tissue, background, and lung tissue).^13^

The lack of bone attenuation information in the DIXON-attenuation correction (DIXON-AC) maps gives rise to average quantification errors of 2% for tissues in the vicinity of osseous structures for whole-body oncological scans.^14^,^15^ On the other hand, myocardial examinations are not affected by missing bone attenuation, as shown in dual-time-point examinations and simulations of PET/CT examinations.^16^–^18^

However, respiratory misalignment between the AC maps and the PET-emission data is a major challenge in myocardial PET-examinations, which causes false-positive findings in up to 40% of the scans.^19^–^21^ Increased frequencies of misalignment artifacts are expected in simultaneous cardiac PET/MRI examinations, due to the requirements of breath-hold for the MR-acquisitions.^22^ Finally, metallic implants, such as cardiac stents or sternal clips after bypass surgery, may alter the magnetic field locally, which may yield artifacts in the MR-AC maps.^23^

The aim of this study was to investigate the frequency and the test–retest reproducibility of artifacts in MR-AC maps acquired as part of PET/MRI imaging protocols and to assess the impact of these distortions on the quantitative accuracy of PET-based perfusion and metabolism measurements in a cohort of patients with advanced coronary artery disease and heart failure.

## Materials and Methods

This retrospective study was approved by the local ethics committee at the Medical University of Vienna.

### PET/MR Imaging

**Figure 1:**
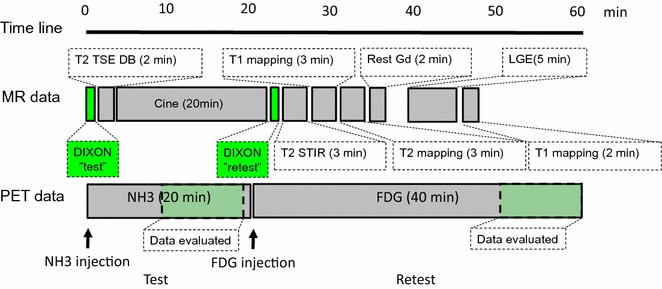
Flow-chart of dual-tracer PET/MRI protocol. Patients were injected with NH_3_ (813 ± 86) MBq with a simultaneous cine-acquisitions (MRI) to assess myocardial functionality. Re-injected with FDG (335 ± 38) MBq, the patients were examined for myocardial viability, with simultaneous acquisitions of myocardial viability through assessments of rest and late-gadolinium enhancement image-acquisitions

Twenty patients scheduled for myocardial viability examinations prior to planned revascularization procedures were enrolled in a combined PET/MR imaging protocol using a fully-integrated PET/MR system (Siemens Biograph mMR^24^). Myocardial viability was accessed using MPI employing ^13^N-NH_3_ (813 ± 86) MBq, and myocardial metabolism using ^18^F-FDG (335 ± 38) MBq (Figure 1, Table 1). All patients underwent a fasting period of 4 h prior to the ^18^F-FDG-PET examination. Following tracer injection, the patients were administered Acipimox (250 mg) to stimulate myocardial tracer uptake of ^18^F-FDG.

**Table 1:** Baseline of the 20 patients (13 Male—M, 7 Female—F) enrolled in this study

Patient #	Gender	Age (years)	Weight (kg)	Injected activity (MBq)		Stent (year)	Sternotomy (year)	Diabetes mellitus
				NH_3_	FDG			
1	F	72	62	620	250		1991, 2011	X
2	M	68	95	865	345	2013		
3	M	57	75	788	345			
4	M	55	73	758	297		2015	
5	F	73	55	881	319			
6	M	67	98	780	329	2013		
7	M	76	90	790	386			
8	M	74	83	782	360			
9	M	76	124	1100	365		2015	
10	F	70	100	868	356		2012	
11	M	75	83	749	400			
12	F	68	90	692	334		2015	
13	F	56	84	813	309			
14	M	73	98	945	362			
15	F	78	94	803	286			
16	M	76	60	822	317			
17	M	47	105	727	308	2014		
18	M	67	64	750	338	2001		X
19	F	62	75	850	317			
20	M	89	62	728	321			

The two PET scans were acquired in listmode, starting at the time of tracer injection (duration: ^13^N-NH_3_: 20 minutes, ^18^F-FDG: 40 minutes). Two DIXON-AC maps were acquired for attenuation correction; one for each acquisition (test-retest), without repositioning of the patient (Figure 1). Given the dual PET and MR imaging protocol were all patients examined using MR body-coils to facilitate optimal MR-imaging quality. The MR body-coils were tightened around the patient torso, thus, minimizing the risk of patient readjustments during the acquisition protocol.

Static PET reconstructions, with and without AC, were reconstructed using an ordered subset expectation maximization (OSEM) algorithm employing 3 iterations, 21 subsets, followed by 5 mm Gaussian smoothing (^13^N-NH_3_: (8-18) minutes p.i., ^18^F-FDG: (30-40) minutes p.i) to evaluate the effect of the AC maps.

### Evaluation of Attenuation Correction Maps

**Figure 2:**
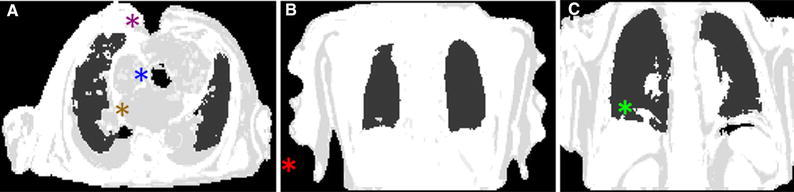
Examples of most frequently observed artifacts on MR-AC (Table 2): **A** Susceptibility artifacts caused by sternotomy (STN, purple asterisk), aortic valve replacement (SMA, blue asterisk), and soft-fat tissue inversion (FSTI, orange asterisk). **B** Truncation artifacts (Tr, red asterisk) were found for all MR-AC maps. **C** Patient with respiratory artifacts (PMA, photopenic type) caused by respiration during the MR-AC acquisition (green asterisk)

Two experienced imaging experts evaluated all 40 DIXON-AC maps (Figure 2) for image artifacts and misalignment of the DIXON-AC maps with the corresponding emission data.

#### Artifacts in the AC map

Test–retest assessment of the image artifacts was performed for all patients, using four artifact categories and combinations thereof (Table 2). Three artifact types were subdivided into two subcategories; susceptibility artifacts: patients with sternotomy (STN) and patients with stents and other metallic artifacts (SMA), tissue inversion artifacts (TI): artifacts caused by inversions of lung and soft tissue (LSTI) as well as of soft and fat tissues (FSTI), respiratory misalignment artifacts: photopenic type artifacts observed at the diaphragm (PMA) and misalignment artifacts of the MR-AC maps and the emission data (Table 2, Figure 2).^25^,^26^

**Table 2:** Categorization of image artifacts on MR-AC: image artifacts arising susceptibility effects, truncation, tissue misclassifications during the calculation of the AC maps (TI), and patient related artifacts

Artifact type	Origin	Effect on the MR-AC maps	Effect on the PET images
1. Susceptibility type artifacts			
Sternotomy (STN)	Metallic sutures after sternotomy	Soft and Fat tissues surrounding the sutures are misclassified as air ATN values in the DIXON-AC maps	Local misclassification of the activity in areas close to the sutures
Metallic implants (SMA)	Metallic implants (stents, aortic valve replacement etc.)	Soft and Fat tissues in the surroundings of the metal-implants are misclassified as air ATN values in the DIXON-AC maps	Local misclassification of the activity in areas close to the implants
2. Truncation			
Truncation (Tr)	Limited field-of-view of the MR-system	Missing tissues in the arm, caused by the limited field of view of the MR system	Local activity misclassifications in the arms
3. Tissue inversion			
Lung/soft tissue (LSTI)	Misclassification of the lung ATN values introduced during classification of soft tissue and lung tissue based on the DIXON-VIBE sequence	Lung tissues are assigned to soft tissue-ATN values	False positive increased lung activity estimates in the areas covered or adjacent to the artifact
Fat/soft tissue (FSTI)	Misclassification of the fat and soft tissue in the fat-water sequences in the DIXON-VIBE acquisition protocol	Inversions of the soft and fat tissues in the MR-AC maps	Erroneous activity estimates in the affected areas
4. Respiratory artifacts			
Misalignment artifact (PMA)	Breathing during the MR-AC acquisition	Soft tissue artifacts observed above the diaphragm	False positive increased lung activity estimates in the vicinity of the misclassification

Of note, susceptibility, and TI artifacts were subdivided into two groups each

#### Respiratory misalignment of PET-emission data and AC maps

MR-AC maps and nonAC-PET images were evaluated for spatial misalignment artifacts through visual inspection using the 3D application integrated into the working suite on the PET/MR console. Manual, rigid co-registrations of AC maps were performed in cases of visible misalignment.

The lung volumes were analyzed for the test–retest setup in all patients by estimating the number of voxels with a lung ATN-value (0.0224 cm^−1^) multiplied with the voxel volume of the acquired DIXON-AC maps (2.6 × 2.6 × 2.6 mm^3^).^12^

#### Corrections

Susceptibility artifacts induced in the sternum and close to the heart were corrected by filling the erroneously segmented tissues with tissue-ATN values corresponding to the tissue-segmentations surrounding the artifact. The corrections were performed using an in-house developed MatLab script (Mathworks, USA), in which the new ATN values were based on the most frequent tissue classification in the six adjacent voxels. The corrected AC maps were stored for subsequent analyses. In addition, the updated AC maps underwent further corrections for truncation artifacts using the MLAA-algorithm implemented in the PET-system (VB-20 version), thus, creating a new series of corrected AC maps. Corresponding AC-PET images were reconstructed for all AC maps using the same reconstruction parameters as for the original reconstruction.

#### Evaluations

All AC-PET images were evaluated in a blinded setup by an experienced nuclear cardiology expert using a clinical software toolbox (Cedars-Sinai QPG/QGS 2013 version, Cedars-Sinai Medical Center, CA, USA). We report scores for the defect extent as well as scar and hibernating tissues obtained in the clinical evaluation software using the standard 17-segment polar plot, as well as the relative differences obtained for the PET-image reconstructions employing the acquired and corrected AC maps, respectively. Furthermore, assessments of image-quality and the clinical impact of using the original AC maps with artifacts were performed in side-by-side comparisons of the AC-PET images. Image quality was evaluated by visual inspection only, whereas clinical evaluations were performed by the assessment of image-quality and the clinical scoring of 17 segment polar maps.^27^

Two 5-point grading scales were used to evaluate the effect of the artifacts; both relating to the AC-PET images reconstructed using the acquired AC maps. Image quality was assessed with the scores: (1 = bad, 2 = poor, 3 = equivocal, 4 = good and 5 = very good), whereas the clinical assessment was based on the criteria: 1 = false-negative finding (certain), 2 = false-negative finding (assumed), 3 = equivocal, 4 = false positive finding (assumed), 5 = false positive score (certain).

Relative difference (RD) maps were calculated for each image reconstruction using Eq. 1,

1 $$ {\text{RD}} = \left( {\frac{{{\text{PET}}_{{{\text{AC}} - {\text{CORRECTED}}}} }}{{{\text{PET}}_{{{\text{AC}} - {\text{ORIGINAL}}}} }}} \right)\; \times \;100\% $$

where PET_AC−CORRECTED_ and PET_AC−ORIGINAL_ represent the reconstructed PET images using the corrected AC maps and the original AC maps, respectively. Average and maximum differences within the myocardium were calculated using a 42% threshold segmentation of the myocardium.^28^,^29^ The effect of the misalignment and the artifacts were evaluated through paired t-tests assuming non-Gaussian distributions, using a significance level of 5% (Graphpad prism 6.0). Both global and regional differences were tested for using a 42% threshold segmentation (SUV_mean_) and a maximum standardized uptake value (SUV_max_) analysis within the segmented myocardium, respectively.

## Results

### Artifacts in the DIXON-AC Images

**Figure 3:**
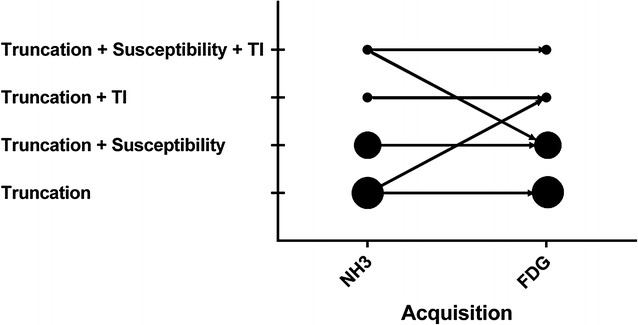
Artifact types (Table 2) observed in the test-rest analysis of the DIXON-AC maps for the individual patients. Circle size corresponds to the number of observations of the given artifact combination for the respective acquisitions (Table 3)

Susceptibility artifacts caused by sternotomy or other metallic objects were observed in 10 (50%) patients (Table 3). Test–retest assessment of artifacts revealed repeated observations of truncation and susceptibility artifacts caused by sternotomy, whereas other artifacts were observed more irregularly (Figure 3).

**Table 3:** Frequency of image artifacts as seen in the DIXON-AC maps for the 20 patients (Table 2)

Artifact type	Patients affected (^13^N-NH_3_ scan) “test”	Patients affected (^18^F-FDG scan) “retest”	Patients affected combined
1. Susceptibility type artifact			
Sternotomy (STN)	4 (25%)	4 (25%)	4 (20%)
Metallic implants (SMA)	6 (30%)	5 (25%)	6 (30%)
2. Truncation			
Truncation (Tr)	20 (100%)	20 (100%)	20 (100%)
3. Tissue inversion			
Tissue inversion (LSTI)	1 (5%)	0 (0%)	1 (5%)
Tissue inversion (FSTI)	2 (10%)	1 (5%)	2 (10%)
4. Respiratory artifacts			
Misalignment artifact (PMA)	7 (35%)	7 (35%)	10 (50%)

Variances in detection of certain artifact types (susceptibility, tissue inversion and respiratory misalignment) were observed in this study (Figure 3). The combined effects denote the collective number of patient affected by the respective artifact types

**Figure 4:**
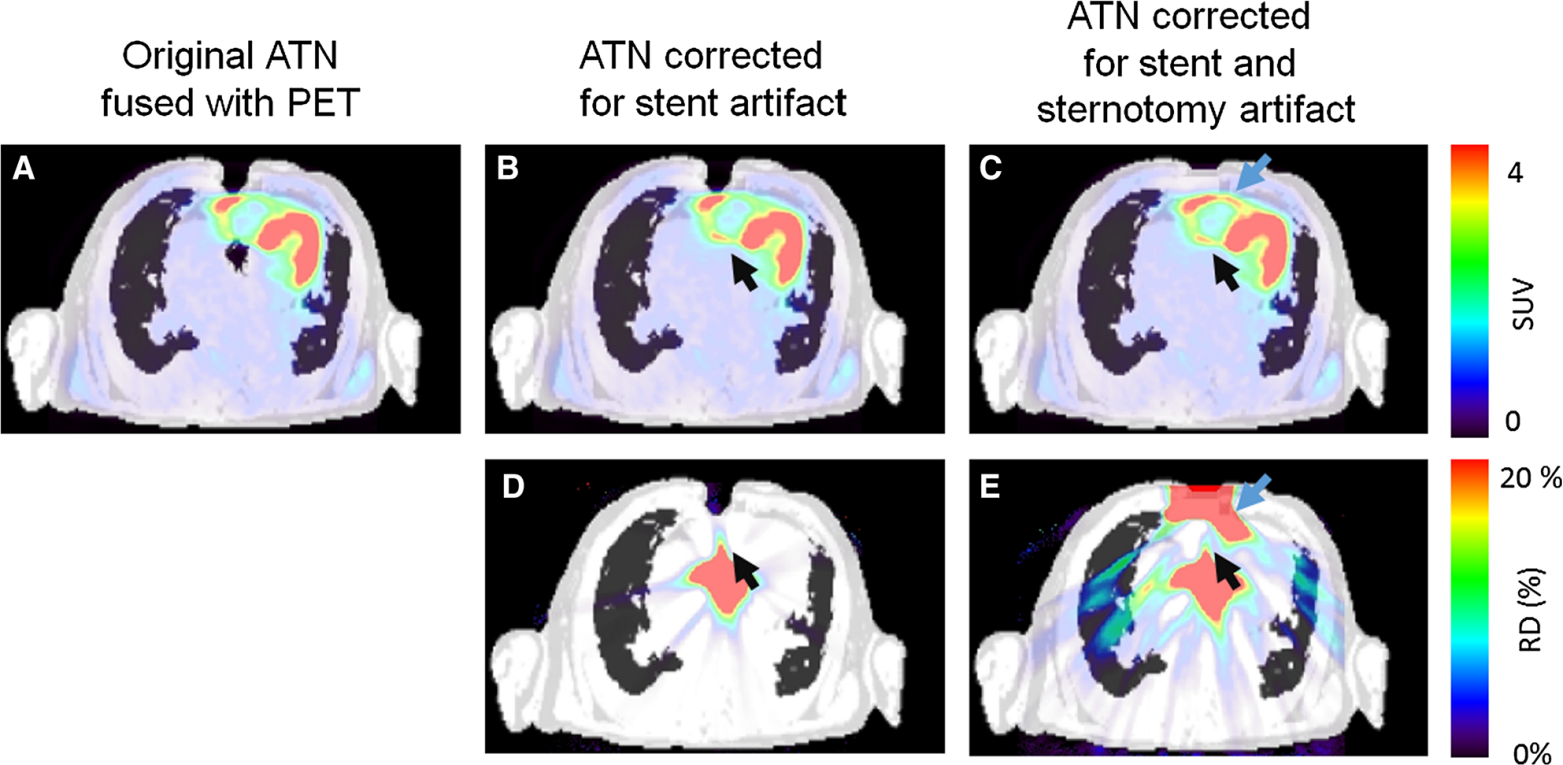
Patient 10 with two categories of susceptibility artifacts caused by sternotomy and an aortic valve replacement (blue and black arrows, respectively). The acquired DIXON-AC map (**A**) was corrected using standard tissue classifications to correct the artifact caused by the valve replacement (**B**) and both stent and sternotomy artifacts (**C**). Relative differences maps were calculated for the reconstructed PET images using the corrected AC maps (**D**, **E**). Significant improvement of the delineation of the right ventricle was observed when correcting the valve replacement artifact (black arrow, **B**-**E**) and both artifacts (blue arrow, **C** and **E**)

**Figure 5:**
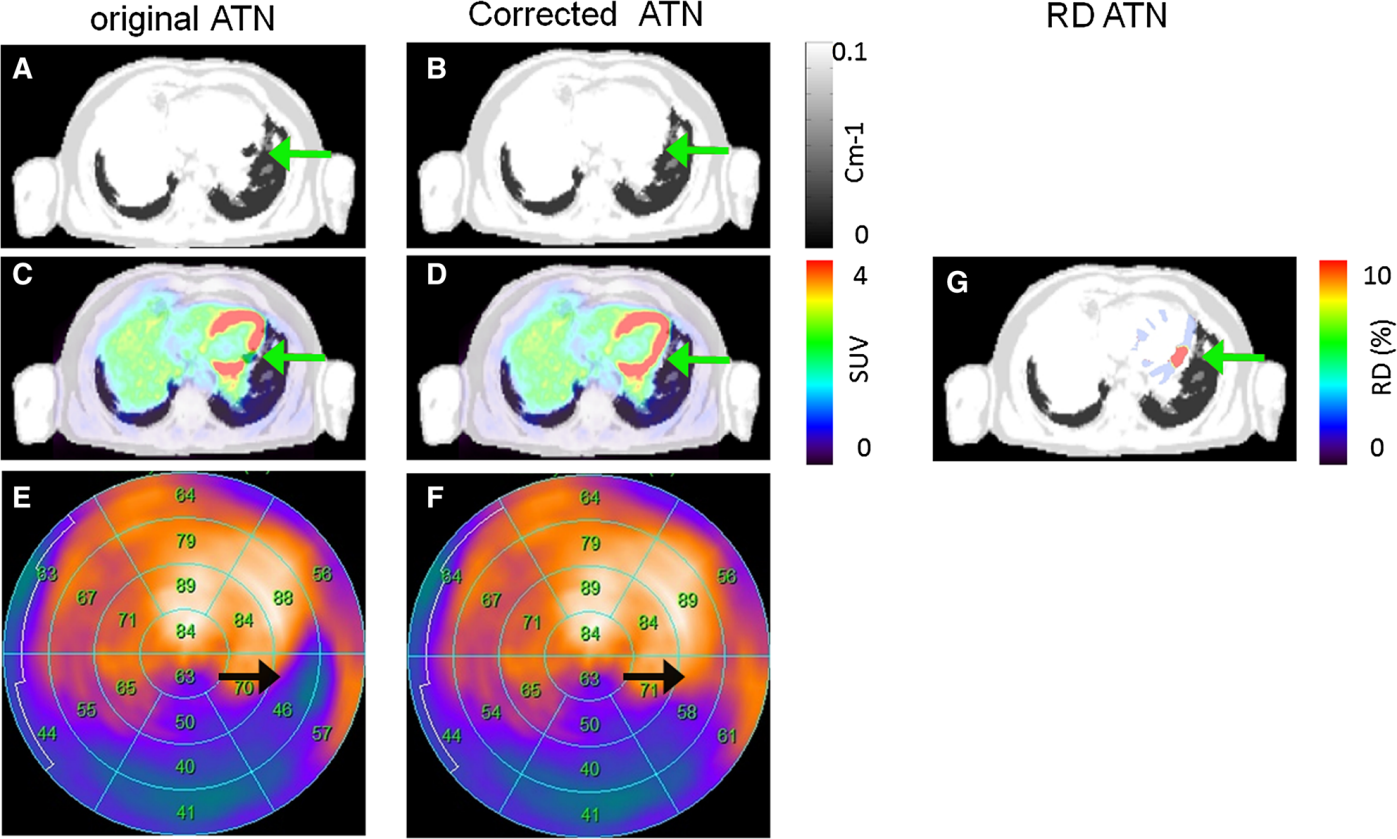
Patient 13 with susceptibility artifact in the inferior wall caused by a stent and corresponding FDG PET reconstructions. Susceptibility artifact in the left circumflex artery was observed in the original AC map (**A**, arrow). Correction of the susceptibility artifact (**B**) changed the interpretation from reduced metabolism to normal metabolism (**C**-**F**, arrows). The susceptibility artifact accounted for relative differences of more than 10% in the affected region (**G**)

Extensive tissue-misclassification artifacts, based on soft/fat tissue and air inversion, were observed in patients following sternotomy (STN) (Figure 2). Susceptibility artifacts resulted in regional underestimation of the tracer-uptake (Figures 4 and 5). Retrospective correction of the susceptibility artifacts led to increased activity levels in the myocardium of 2%, whereas local differences of more than 100% were observed (Table 4).

**Table 4:** Relative differences (%) observed in the myocardium when correcting the artifact in the original AC maps

Artifact type	Effect on NH3 acquisitions		Effect on FDG acquisitions	
	Average	Maximum	Average	Maximum
Susceptibility				
Sternotomy (STN)	2 ± 1	11	2 ± 1	13
Metallic implants (SMA)	1 ± 1	53	2 ± 2	196
Truncation				
Truncation (Tr)	10 ± 8	84	11 ± 4	66
Tissue inversion				
Tissue inversion (FSTI)	26	431	N/A	N/A
Respiratory misalignment				
Respiratory misalignment	2 ± 4 (*P* = .03)*	221	6 ± 7 (*P* = .002)*	291 (*P* = .005)*
Combined effects				
Respiratory misalignment, susceptibility, and Truncation	10 ± 9	270 (*P* = .008)*	17 ± 12	333 (*P* = .002)*

Average effects of the SUV_mean_ were calculated for the entire myocardium, using a 42% threshold segmentation, while maximum relates to the single most changed voxel. Artifact types not mentioned in this table were not considered for evaluation. *Indicate statistically significant differences

Tissue misclassifications caused by stents (SMA) in the coronary arteries accounted for local differences of over 100% (Figure 5). Assessment of the myocardial function in a clinical evaluation tool indicated a hypo-metabolic region for the left circumflex artery in the PET images following reconstruction with the original AC map (Figure 5D). Manual correction of the artifact led to a significantly improved uptake in the affected segments with a relative increase of 12 points (Figure 5D, E), which was then interpreted as normal uptake following false-positive reading of the original data.

Truncation artifacts (Tr) were observed in 100% of the patients (Table 3). Corrections for truncation artifacts using a MLAA-based AC maps yielded global biases of up to 20% in the myocardium (Table 4).

Soft tissue/fat tissue inversions (FSTI) were observed for one patient (patient 9, Figure 2A) in one of the acquisitions (^13^N-NH_3_). The tissue-misclassification led to a RD of 25% in the myocardium, when compared to reconstructions using the registered, non-inverted DIXON-AC map.

### Respiratory Misalignment of PET-Emission Data and AC Maps

**Figure 6:**
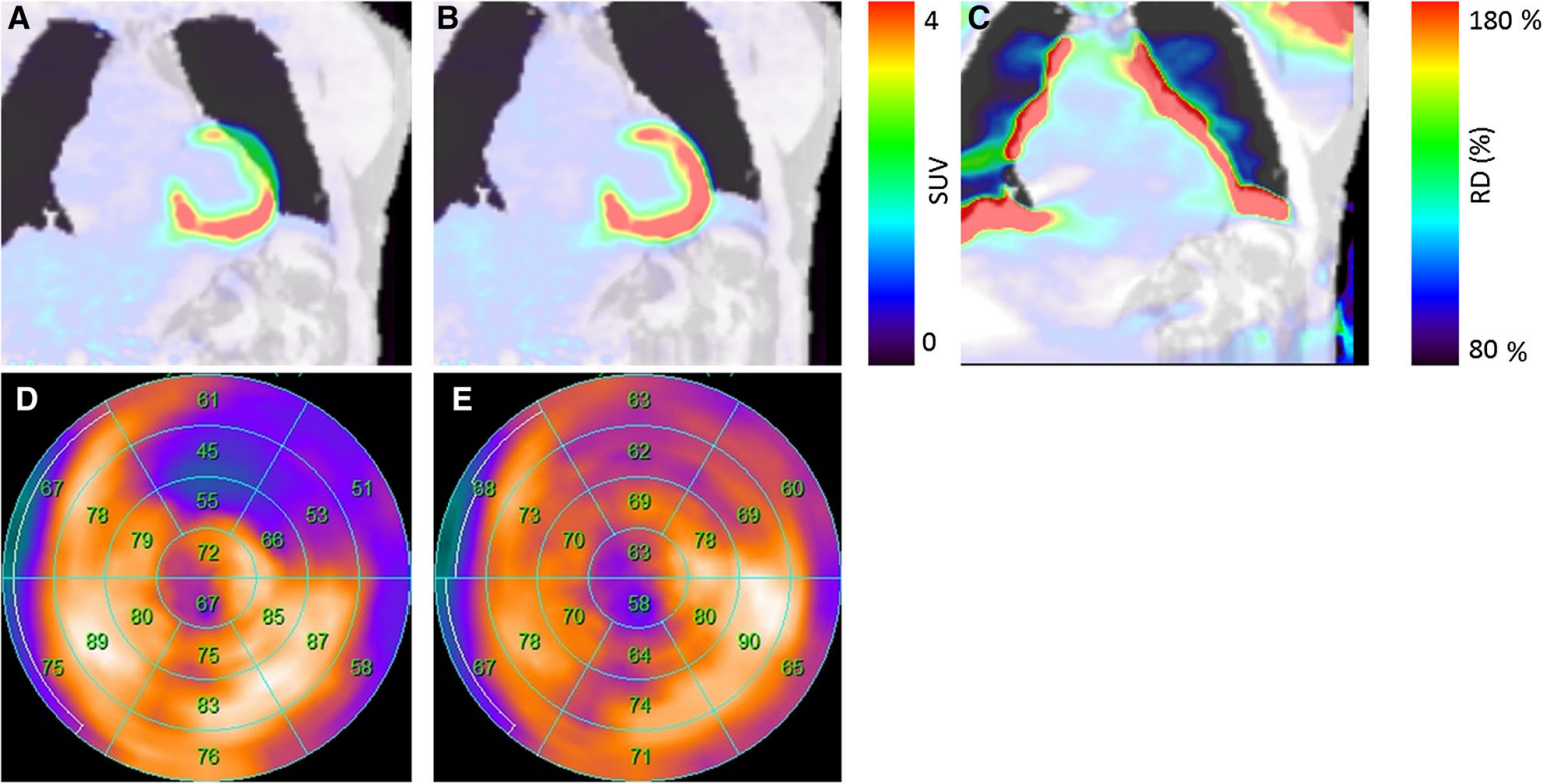
**A** Axial misalignment of 18 mm was observed between the PET-emission data and the DIXON-AC map for patient 14. **B** Rigid co-registration of the AC map subsequently resulted in normal uptake in the anterior wall. **C** Activity changes of more than 80% in were observed in the relative difference map, with the PET data reconstructed using the standard AC map serving as reference. Corresponding polar-map analyses revealed changes in size and severity of hypo-metabolic area for the misaligned AC map (**D**) and the re-aligned map (**E**)

Misalignment (Figure 6) of the acquired AC maps and PET-emission data was observed in 18 of the 20 patients (Table 5). Average misalignment across all patients was (7 ± 4) mm (range: (−18 to 12) mm), using the nonAC-PET image position as reference point. Severe underestimation of the myocardial uptake was observed in cases of misalignment >10 mm (Figure 6). Correction of the alignment resulted in changes of up to 291% of the reconstructed activity within the anterior wall (Table 3, Figure 6C). The changes in the reconstructed activity resulted in a RD of 17 points in clinical evaluation tools for the mid-anterior segment (Figure 6D, E), recovering the false-positive finding.

**Table 5:** Frequency and effect of the misalignment between the PET-emission data and the AC maps (*n* = 20)

Respiratory misalignment (offset)	^13^N-NH_3_ scan	^18^F-FDG scan	Patients affected combined
Number of patients	11 (55%)	14 (70%)	18 (90%)
Offset (mean ± std) [mm]	8 ± 4	7 ± 4	7 ± 4

Average offsets are reported in the axial direction only

### Test–Retest of DIXON-AC Maps

The test–retest of the patients revealed differences in the frequency of the artifacts reported (Figure 3). One patient (#10) had one TI-AC map (^13^N-NH_3_) that was not reproduced (retest). Reconstructions of the PET-emission data using a correct tissue-classified AC map resulted in a RD of 26% of the obtained activity within the myocardium (Table 4).

Pooled analyses of the lung volumes for the two acquisitions revealed consistent patient cooperation during the AC acquisitions (^13^N-NH_3_: (2.6 ± 0.7) L, FDG: (2.7 ± 0.7) L), although with great intra-patient variations in the lung volume (ratio: 1.0 ± 0.2, range: 0.6-1.4). Five patients were found to have incoherent lung volumes (intra-scan variation of more than 10%) caused by breathing during the MR attenuation correction map acquisition.

### Clinical Assessment of PET Reconstructions Using the Corrected/Non-corrected AC Maps

Three patients (15%) had false-positive findings when employing the uncorrected MR-AC maps: patient 10: TI, sternotomy, aortic valve replacement (varia) and respiratory misalignment (type 2) (Figure 4), patient 13: susceptibility artifact caused by stent (Figure 5) and patient 14: misalignment of the AC map and the emission data (Figure 6). Re-assessment of the patients with corrected MR-AC maps revealed normal uptake in the affected areas. No significant changes in the clinical assessment were observed in the other 17 patients.

Image-quality was reported to be equivocal in the myocardial region for 16 patients who had no substantial effect of artifacts in the AC maps. Four patients were reported to have improved image quality, one had improved image quality (score 4, 1 patient), whereas three patients were evaluated as having significantly improved image quality (score 5, 3 patients) for reconstructions employing the corrected AC maps.

Evaluations of the defect extent as well as the percent-wise left-ventricular occupation of the scar and hibernating tissues revealed nonsignificant effects of the artifacts in 17/20 (85%) of the patients (Table 6). However, considerable differences were observed in 3 patients (# 10, 13, 14), with relative changes of 60% to 100% in the in the clinical evaluation following the correction of the AC maps (Table 6).

**Table 6:** Clinical scores obtained for the defect extent as well as scar and hibernating tissues obtained in a clinical evaluation software

(%)	Acquired	Corrected	Corrected + MLAA
Extent			
Mean ± SD	26 ± 12	25 ± 12	21 ± 12
RD (range)	−3 ± 21(−58:33)		−17 ± 19 (−58:5)
Scar			
Mean ± SD	13 ± 10	13 ± 9	11 ± 8
RD (range)	10 ± 35 (−20:100)		−7 ± 31 (−52:50)
Hibernating			
Mean ± SD	8 ± 8	6 ± 7	5 ± 7
RD (range)	−11 ± 31 (−80:33)		−30 ± 30 (−80:0)

Nonsignificant changes were reported for the grouped analyses, though noteworthy differences of up to 100% were observed for three patients with reported changes in the image-quality (Figures 4, 5 and 6)

## Discussion

The aim of this study was to evaluate the frequency of artifacts in standard DIXON-AC maps and their effect in myocardial PET/MR imaging in a cohort of patients with consecutive heart failure, where metallic implants, such as from sternotomy or stents are a cause of susceptibility artifacts. Our main finding is that clinical analyses of cardiac perfusion or metabolism can be affected by the presence of severe distortion of MR-AC maps.

Susceptibility and TI artifacts of the AC maps were observed in 10 of 20 patients (50%). Of those, we observed susceptibility artifacts in 16 of the 20 test–retest acquisitions, TI for 6 acquisitions and misalignment artifacts (PMA) in 14 acquisitions. In addition to the AC image artifacts, a high frequency of spatial misalignment was observed in this study (90% cases). This is ascribed to the recommendations by the vendor of acquiring the MR-AC maps in expiratory breath-hold.^30^ The many breath-holds during standard cardiac MR acquisitions will cause fatigue in the patients, which will alter the lung volume during the acquisition protocol. Together with the 19 seconds acquisition time for the DIXON-AC series, acquired in expiratory breath-hold, this protocol can be challenging to clinical patients.^16^ The impact of these artifacts is expected to be significant for the calculation of the myocardial perfusion, given the non-linear nature of kinetic modeling.^31^ The misalignment artifacts may be corrected for through acquisitions of the DIXON-sequence in several respiratory phases,^32^,^33^ or by a subsequent DIXON-acquisition at the end of the examination.

Susceptibility artifacts can be corrected retrospectively by filling the artifactual region of the DIXON-AC maps with tissue-ATN coefficients obtained from the adjacent voxels.^34^–^37^ Misalignment artifacts, on another hand, can be corrected retrospectively using manual co-registrations of the AC maps and the emission data.^19^,^20^ Tissue inversion, however, is more challenging to correct.^26^

Regional differences of over 100% within the myocardium were observed when correcting the misclassified tissues to the expected tissue classification (Table 4). Despite substantial RD, only three patients (15%) had changes in the clinical assessment (Figures 4, 5 and 6). This finding correlates with previous findings on misalignment artifacts, which have been reported to be significant in as many as 20% of the myocardial examinations.^19^,^20^ In this study, susceptibility artifacts were reported to have varying effects on the myocardial assessment of the patients, depending on the location of the metallic implants. Artifacts arising from sternotomy had only minor effects on the clinical assessment of the patients, whereas metallic implants in the vicinity of the left ventricular wall had significant impact on the clinical assessment (Figures 4 and 5).

Corrections of the truncation artifacts using the MLAA correction algorithm resulted in a uniform scaling of 10% of the tracer-activity distribution in the myocardium (Table 4) and, thus, did not affect the clinical reading (Table 6).

The reported image-quality was improved for four patients (1 case of score 4 and 3 cases of score 5) following the retrospective correction of the image artifacts. The patient evaluated with score 4 had improved image-quality based on corrections of misalignments, though without changes in the clinical assessment. In the three cases with score 5, the clinical assessment of the patients also changed (patients 10, 13, and 14).

The main limitation of our study is the simplistic nature of the corrections for the susceptibility artifacts. The reason for choosing relatively simple correction methods was to show the effect of the different artifacts in the quantitative properties of the myocardial PET images, as a proof of concept and first approximation. Besides the simple algorithm proposed here, complex algorithms for correction of MR-AC image artifacts have been proposed to correct for the MR-AC image artifacts.^34^,^35^,^37^

A second limitation is that not all image artifacts were corrected in this study. For example, misalignment artifacts (PMA) caused by breathing during MR-AC acquisitions were not compensated for, due to the gradual change of the respiratory phase throughout acquisition. We decided not to include corrections for these artifacts because they usually are located in regions relatively far from the myocardium and are therefore not expected to affect the evaluation of the myocardial uptake. Another limitation of our study was the relatively limited patient cohort examined.

In summary, we report a high frequency of MR-AC artifacts (90%) of all patients examined in this study. Despite the high frequency of artifacts, the rate of false-positive findings was comparable to previous reports.^19^,^20^ The majority of the artifacts did not affect the quantitative assessment of the myocardium, but could question the validity of prospective studies involving patients with cardiac assisting devices.

## Conclusion

The majority of the artifacts observed in the DIXON-AC maps obtained in a clinical PET/MR imaging protocol do not affect the quantitative assessment of the patients. However, the irregular detection of susceptibility artifacts may impact the quantitative accuracy of patients with cardiac assisting devices. Therefore, PET/MRI in cardiac studies mandates the thorough examination of DIXON-AC maps and correction of misalignment and susceptibility artifacts in the vicinity of the myocardium to ensure the best possible diagnostic quality in clinical routine.

## New Knowledge Gained

Misalignment of the attenuation correction maps is a frequently observed problem for myocardial viability studies in PET/MR imaging. Combined susceptibility artifacts and tissue inversion translate into erroneous activity measures in the myocardium and, thus, affect myocardial perfusion estimates. A thorough evaluation of the DIXON-AC map, following a correction of these artifacts, is required to obtain reliable results for cardiac PET/MR examinations.

## Electronic supplementary material

Below is the link to the electronic supplementary material.

Supplementary material 1 (PPTX 1459 kb)

## Abbreviations

AC: Attenuation correction
ATN: Attenuation
CAD: Coronary artery disease
CT: Computed tomography
LCX: Left circumflex coronary artery
MPI: Myocardial perfusion imaging
MRI: Magnetic resonance imaging
PET: Positron emission tomography
SUV_max_: Maximum standardized uptake value
TI: Tissue inversion
