# Supplementary material for: Assessment of attenuation correction for myocardial PET imaging using combined PET/MRI
Source: J Nucl Cardiol. 2017 Nov 22;26(4):1107–18. doi: 10.1007/s12350-017-1118-2 (PMC6660490; doi:10.1007/s12350-017-1118-2)
Supplement: Supplementary file 1 — Supplementary material 1 (PPTX 1459 kb) [file 12350_2017_1118_MOESM1_ESM.pptx]

## Slide 1
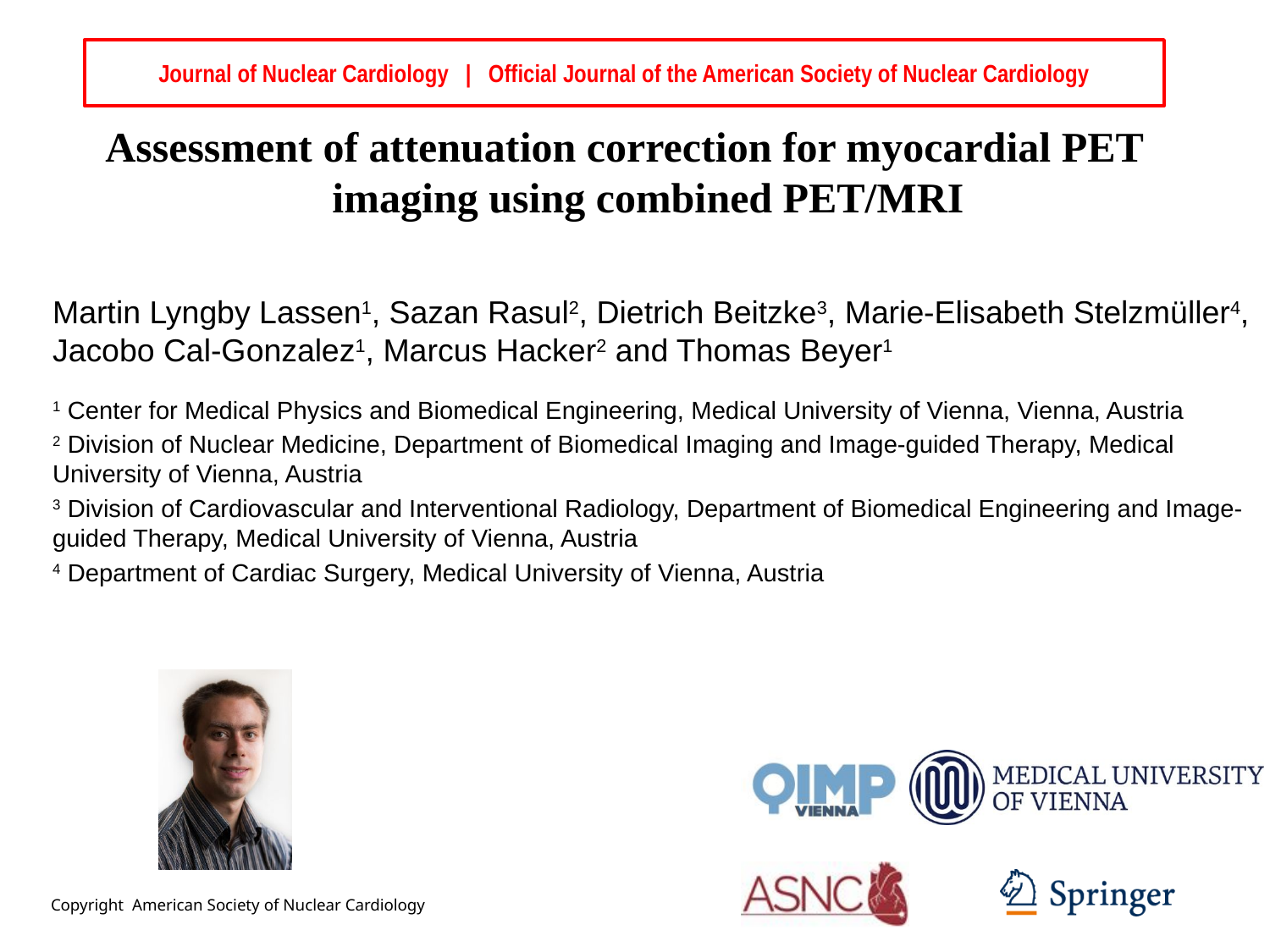

Journal of Nuclear Cardiology | Official Journal of the American Society of Nuclear Cardiology
# Assessment of attenuation correction for myocardial PET imaging using combined PET/MRI
Martin Lyngby Lassen1, Sazan Rasul2, Dietrich Beitzke3, Marie-Elisabeth Stelzmüller4, Jacobo Cal-Gonzalez1, Marcus Hacker2 and Thomas Beyer1
1 Center for Medical Physics and Biomedical Engineering, Medical University of Vienna, Vienna, Austria
2 Division of Nuclear Medicine, Department of Biomedical Imaging and Image-guided Therapy, Medical University of Vienna, Austria
3 Division of Cardiovascular and Interventional Radiology, Department of Biomedical Engineering and Image-guided Therapy, Medical University of Vienna, Austria
4 Department of Cardiac Surgery, Medical University of Vienna, Austria
Copyright American Society of Nuclear Cardiology

## Slide 2
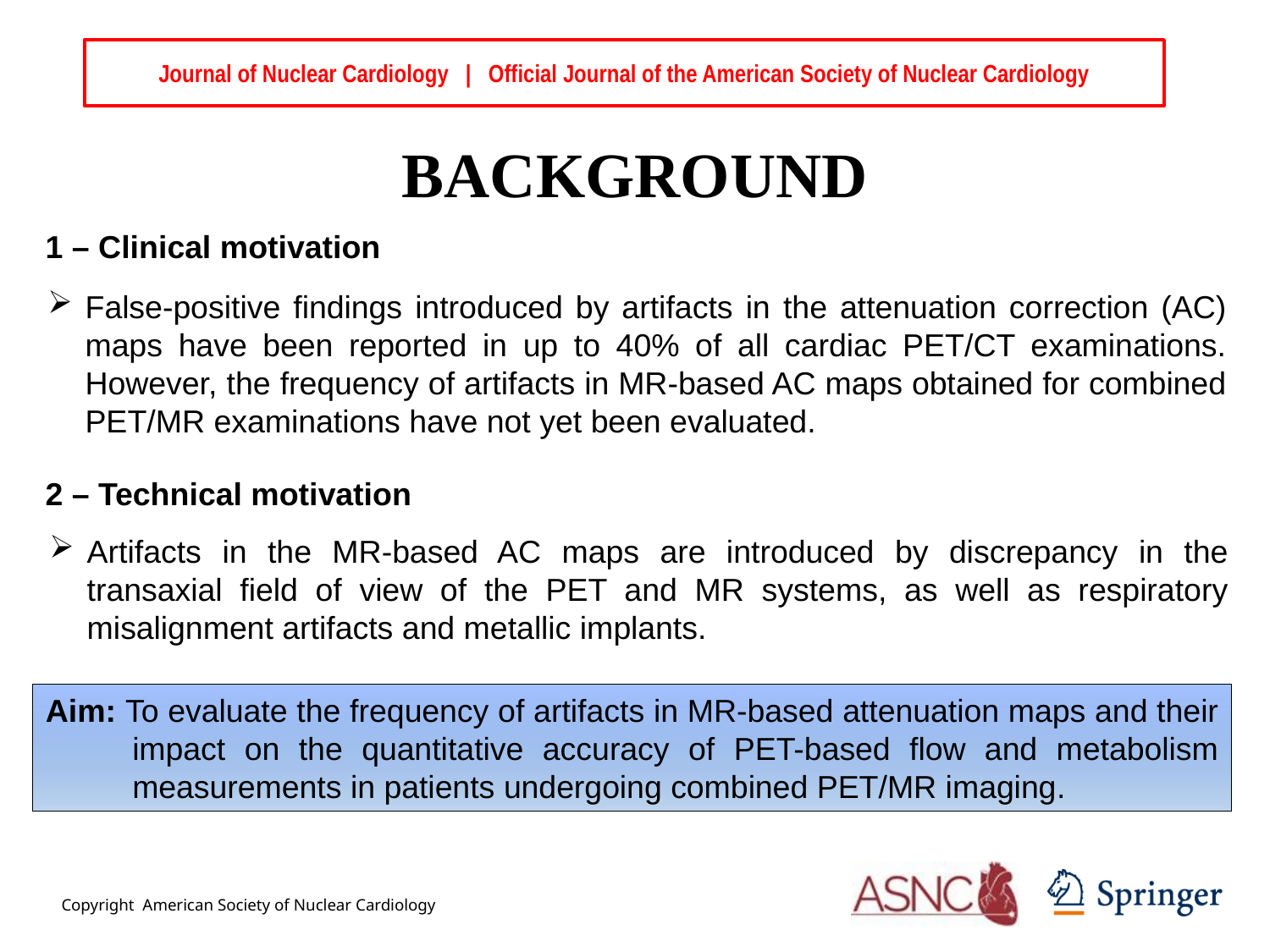

Journal of Nuclear Cardiology | Official Journal of the American Society of Nuclear Cardiology
# BACKGROUND
1 – Clinical motivation
False-positive findings introduced by artifacts in the attenuation correction (AC) maps have been reported in up to 40% of all cardiac PET/CT examinations. However, the frequency of artifacts in MR-based AC maps obtained for combined PET/MR examinations have not yet been evaluated.
2 – Technical motivation
Artifacts in the MR-based AC maps are introduced by discrepancy in the transaxial field of view of the PET and MR systems, as well as respiratory misalignment artifacts and metallic implants.
Aim: To evaluate the frequency of artifacts in MR-based attenuation maps and their impact on the quantitative accuracy of PET-based flow and metabolism measurements in patients undergoing combined PET/MR imaging.
Copyright American Society of Nuclear Cardiology

## Slide 3
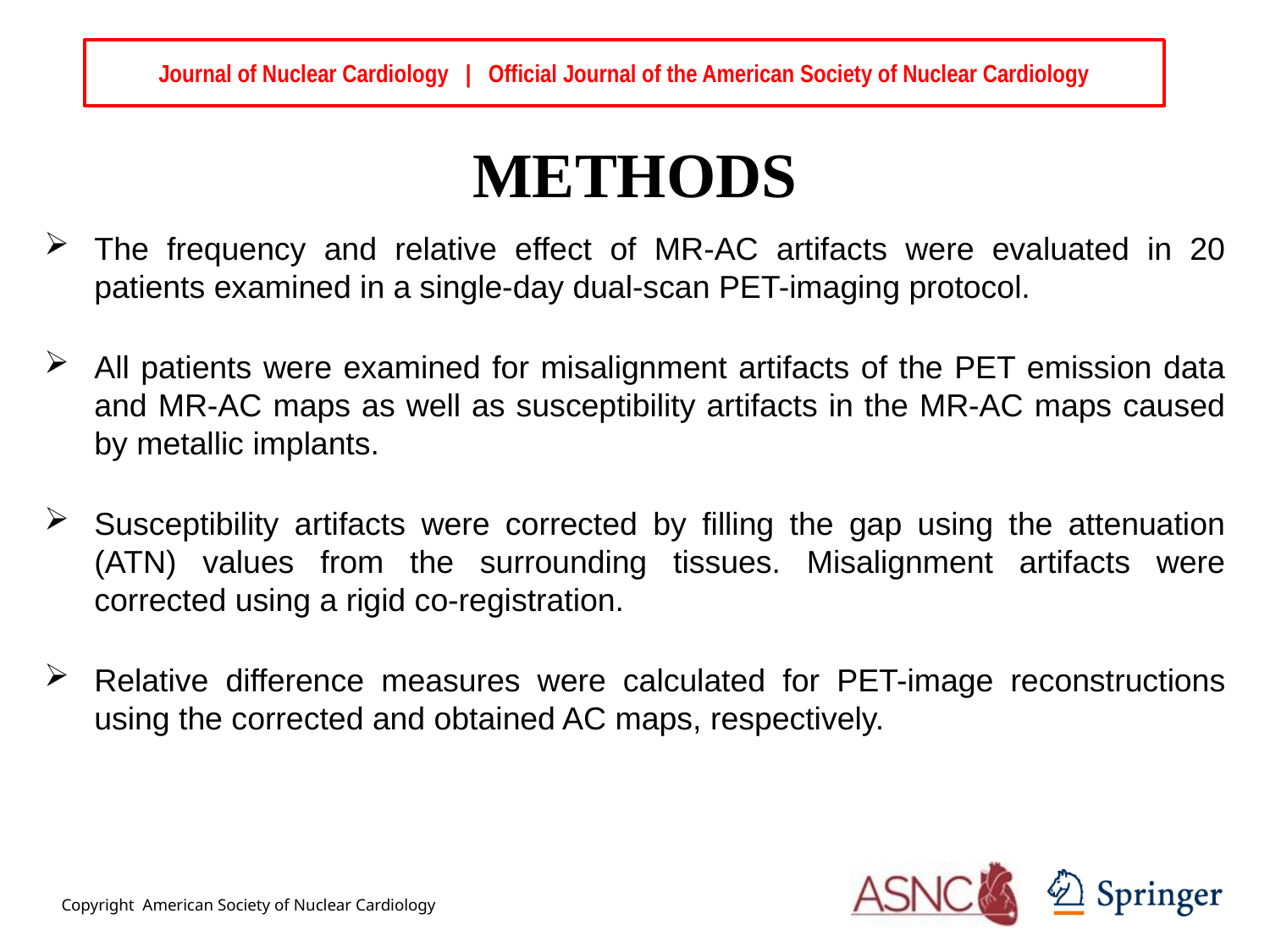

Journal of Nuclear Cardiology | Official Journal of the American Society of Nuclear Cardiology
# METHODS
The frequency and relative effect of MR-AC artifacts were evaluated in 20 patients examined in a single-day dual-scan PET-imaging protocol.
All patients were examined for misalignment artifacts of the PET emission data and MR-AC maps as well as susceptibility artifacts in the MR-AC maps caused by metallic implants.
Susceptibility artifacts were corrected by filling the gap using the attenuation (ATN) values from the surrounding tissues. Misalignment artifacts were corrected using a rigid co-registration.
Relative difference measures were calculated for PET-image reconstructions using the corrected and obtained AC maps, respectively.
Copyright American Society of Nuclear Cardiology

## Slide 4
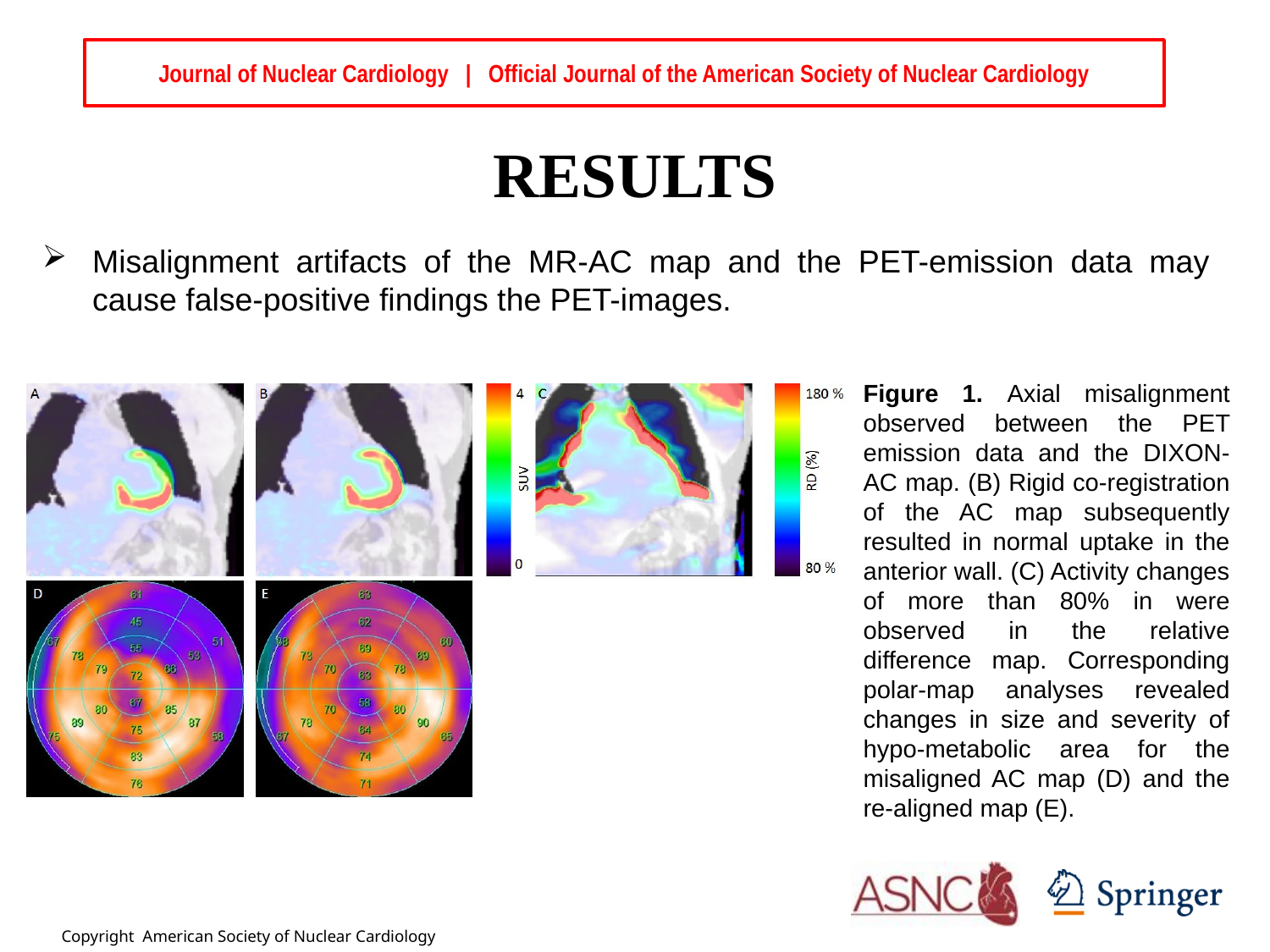

Journal of Nuclear Cardiology | Official Journal of the American Society of Nuclear Cardiology
# RESULTS
Misalignment artifacts of the MR-AC map and the PET-emission data may cause false-positive findings the PET-images.
Figure 1. Axial misalignment observed between the PET emission data and the DIXON-AC map. (B) Rigid co-registration of the AC map subsequently resulted in normal uptake in the anterior wall. (C) Activity changes of more than 80% in were observed in the relative difference map. Corresponding polar-map analyses revealed changes in size and severity of hypo-metabolic area for the misaligned AC map (D) and the re-aligned map (E).
Copyright American Society of Nuclear Cardiology

## Slide 5
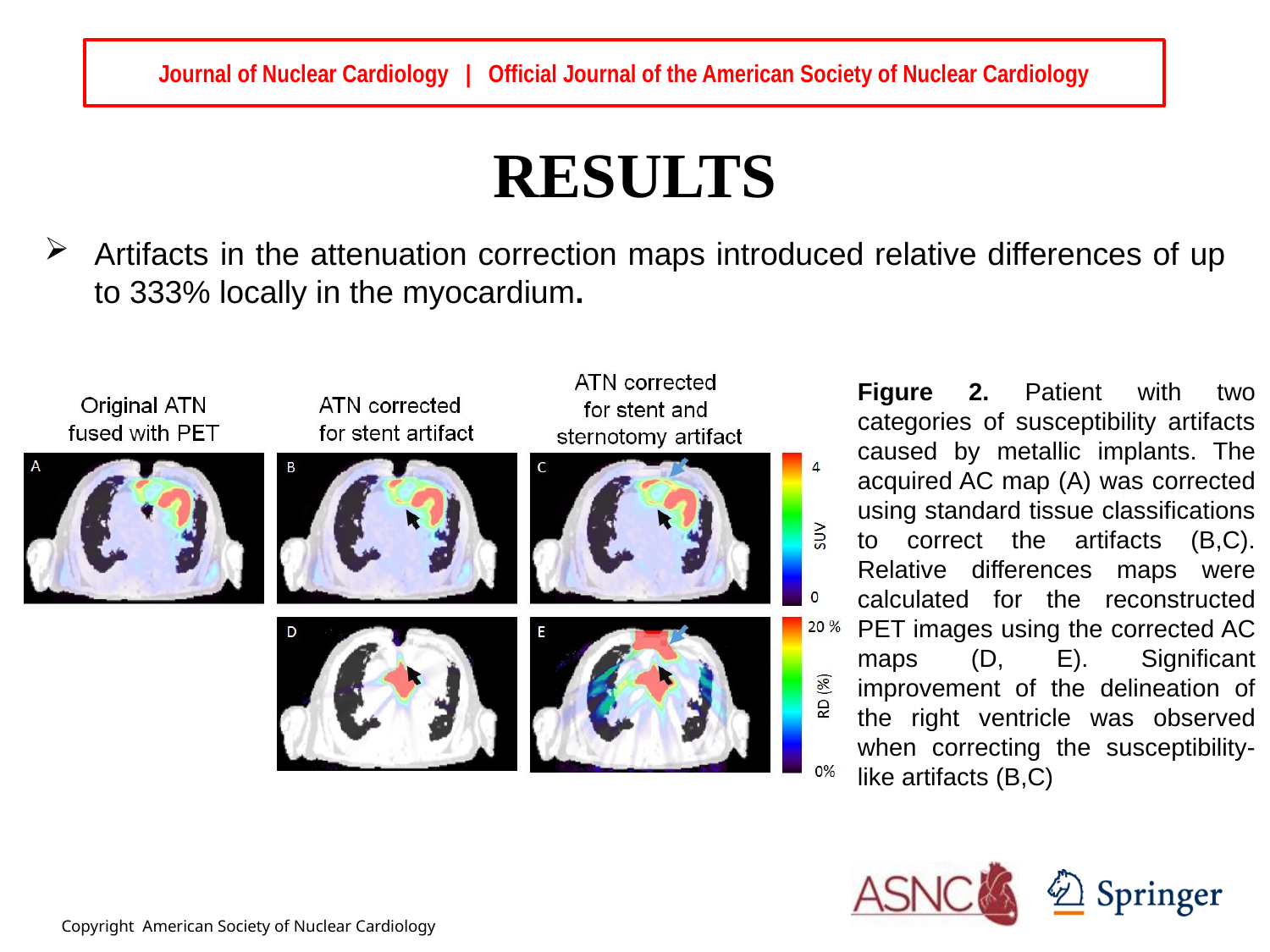

Journal of Nuclear Cardiology | Official Journal of the American Society of Nuclear Cardiology
# RESULTS
Artifacts in the attenuation correction maps introduced relative differences of up to 333% locally in the myocardium.
Figure 2. Patient with two categories of susceptibility artifacts caused by metallic implants. The acquired AC map (A) was corrected using standard tissue classifications to correct the artifacts (B,C). Relative differences maps were calculated for the reconstructed PET images using the corrected AC maps (D, E). Significant improvement of the delineation of the right ventricle was observed when correcting the susceptibility-like artifacts (B,C)
Copyright American Society of Nuclear Cardiology

## Slide 6
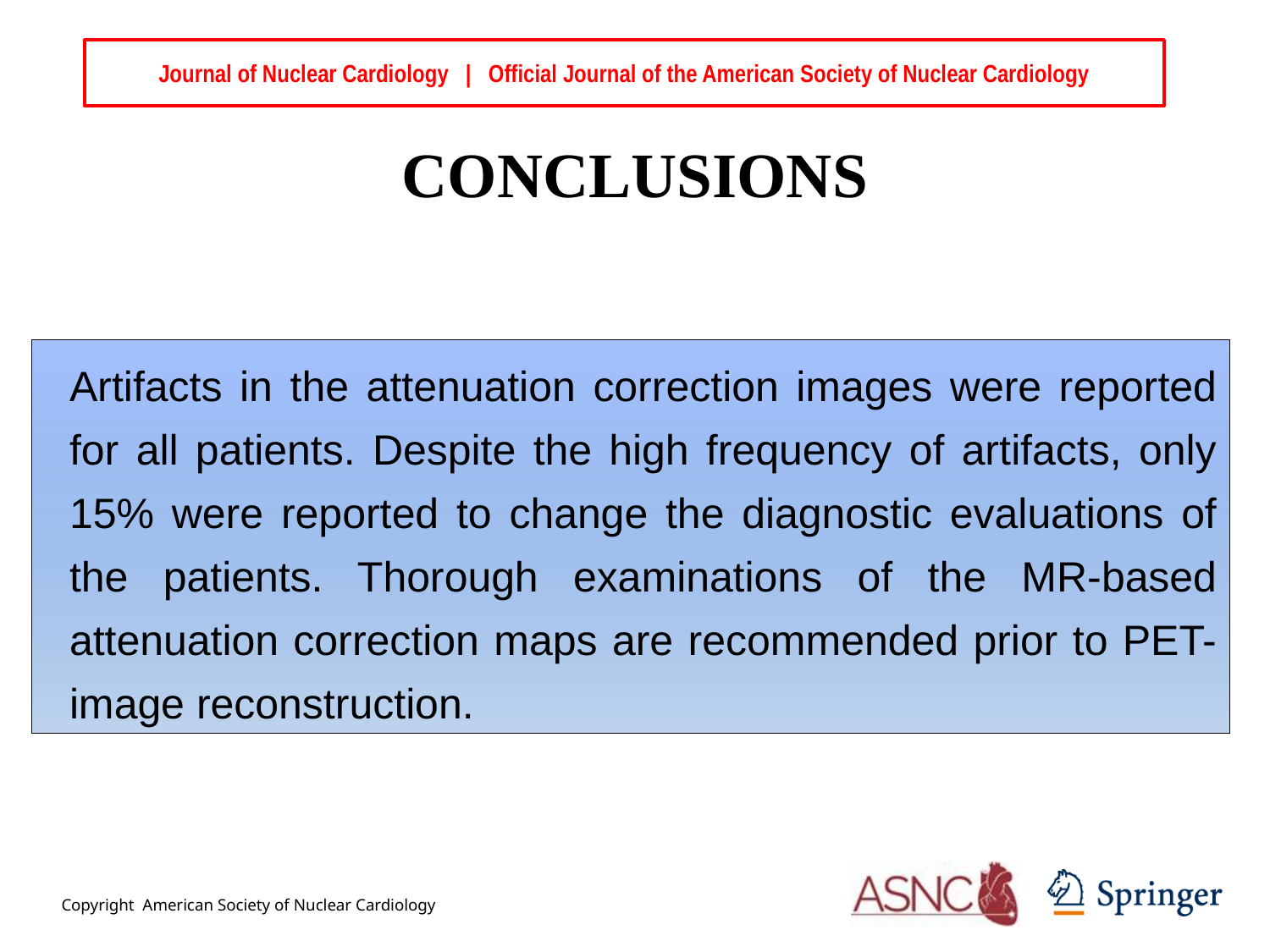

Journal of Nuclear Cardiology | Official Journal of the American Society of Nuclear Cardiology
# CONCLUSIONS
Artifacts in the attenuation correction images were reported for all patients. Despite the high frequency of artifacts, only 15% were reported to change the diagnostic evaluations of the patients. Thorough examinations of the MR-based attenuation correction maps are recommended prior to PET-image reconstruction.
Copyright American Society of Nuclear Cardiology
